# The use of a volatile anesthetic regimen protects against acute normovolemic hemodilution induced myocardial depression in patients with coronary artery disease

**DOI:** 10.4103/0973-6247.44474

**Published:** 2009-01

**Authors:** Sratwadee Lorsomradee, Suraphong Lorsomradee

**Affiliations:** *Department of Anesthesiology, Chiangmai University Hospital, Thailand*

**Keywords:** Acute normovolemic hemodilution, coronary artery disease, volatile anesthetic

## Abstract

**Background:**

Previous studies indicated that acute normovolemic hemodilution (ANH) was associated with a depression of myocardial function in coronary surgery patients with baseline heart rate faster than 90 bpm. It was suggested that this phenomenon could be explained by the occurrence of myocardial ischemia. In the present study, we hypothesized that the cardioprotective properties of a volatile anesthetic regimen might protect against the ANH related myocardial functional impairment.

**Materials and Methods:**

Forty elective coronary surgery patients with baseline heart rate faster than 90 bpm were randomly allocated to receive different anesthetic regimens. Group A (n = 20) received midazolam-based anesthesia. Group B (n = 20) received a sevoflurane-based anesthesia. Five-lead electrocardiogram, pulse oximetry, capnography, radial arterial pressure, and Swan Ganz continuous thermodilution cardiac output via right internal jugular vein were monitored. Measurements were obtained before and after ANH. Data were compared using paired t test. All data were expressed as mean ± SD. Data were considered significant if *P* < 0.05.

**Results:**

After ANH, systemic vascular resistance was slightly decreased in group A while there was a significant decrease in group B. In group A, cardiac output was slightly decreased from 5.07±1.17 l/min to 5.02±1.28 l/min after ANH, whereas in group B, cardiac output was significantly increased from 4.84±1.21 l/min to 6.02±1.28 l/min after ANH.

**Conclusion:**

In coronary surgery patients, with baseline heart rate faster than 90 bpm, anesthesia with sevoflurane during ANH was associated with an improvement in myocardial function after ANH, which was not present in patients anesthetized with midazolam.

## Introduction

Recently, the premise that hemoglobin (Hb) levels should be kept above 9 to 10 g per dL has been challenged by clinical and experimental studies suggesting that acute normovolemic hemodilution (ANH) and restrictive transfusion strategies (Hb target of 7-8 g/dL) in critically ill and surgical patients can reduce blood transfusion requirements without compromising clinical outcome.[1,2] With this technique, the adequacy of tissue oxygenation and organ function is maintained by compensatory increases in cardiac output, improved blood flow distribution, and higher oxygen extraction ratios.[3–5] In the myocardium, hemodilution-induced lowering of blood viscosity is thought to facilitate blood flow through stenotic and collateral vessels, thereby counteracting the reduced blood oxygen-carrying capacity.[6] However, a previous study[7] indicated that acute normovolemic hemodilution (ANH) was associated with a depression of myocardial function in coronary surgery patients with baseline heart rate faster than 90 bpm. It was suggested that this phenomenon would be explained by the occurrence of myocardial ischemia. In the present study, we hypothesized that the cardioprotective properties of a volatile anesthetic regimen[8,9] might protect against the ANH related myocardial functional impairment. To test this hypothesis, we compared the effects of a midazolam-based intravenous anesthetic and a sevoflurane-based volatile anesthetic regimen on hemodynamic and cardiac function after ANH in coronary surgery patients.

## Materials and Methods

The study was approved by the institutional ethical committee, and written informed consent was obtained. Forty patients, with baseline heart rate faster than 90 bpm, undergoing elective coronary surgery with cardiopulmonary bypass (CPB) were included. Inclusion criteria were as follows: a screening hemoglobin concentration > 12 g/dL in men or 11 g/dL in women; stable angina; left ventricular ejection fraction > 30%; and absence of significant coexistent diseases, namely, valvular disease, recent myocardial infarct (< 6 weeks), significant carotid stenosis (> 70%) or recent stroke (< 3 weeks), renal insufficiency (estimated creatinine clearance < 20 mL/min), chronic respiratory disease (arterial oxygen pressure > 7 kPa on room air), liver insufficiency (aspartate transaminase or alanine transaminase two or more times the upper range), and uncontrolled hypertension or diabetes mellitus. All preoperative cardiac medication except for the angiotensin-converting enzyme inhibitors was continued until the morning of surgery. All patients received standard premedication of 7.5 mg oral midazolam 90 min before surgery. In the operating room, all patients received routine monitoring, including five-lead electrocardiography, radial and pulmonary artery catheters with continuous cardiac output measurement (Swan Ganz CCO/VIP; Edwards Lifesciences LLC, Irvine, CA), pulse oximetry, capnography, and blood and urine bladder temperature monitoring.

Patients were randomly allocated to two different anesthetic regimens. Group A (n = 20) received midazolam-based anesthesia. Group B (n = 20) received sevoflurane-based anesthesia. The surgeon, an independent investigator, and the patient were blinded to the type of anesthesia by positioning of sheets covering the vapor, and by connecting a midazolam infusion pump to the intravenous line or a hidden syringe, respectively. After preoxygenation with 100% oxygen, anesthesia was induced by fentanyl (Fentanyl-Janssen, Janssen-Cilag, Beerse, Belgium) 0.01 mg/kg and relaxation with cisatracurium (Nimbex, GlaxoSmithKline, Parma, Italy) 0.2 mg/kg together with midazolam (Dormicum, Roche, Fontenay-sous-Bois, France) or sevoflurane (Sevorane, Abbott). In group A, anesthesia was performed with midazolam based total intravenous anesthesia (TIVA) by constant intravenous midazolam infusion at 1-5 µg/kg/min after a bolus dose of 0.15 mg/kg. In group B, anesthesia was performed with sevoflurane based volatile induction and maintenance anesthesia (VIMA) by mask induction follow by 0.5-2% end-tidal sevoflurane. In both groups, anesthetic depth was adjusted and maintained to keep bispectral index at 40-60.

After anesthesia induction, blood was withdrawn (60 to 80 mL/min) from a central vein by gravity into citrate-phosphate-dextrose collection bags that were placed on a rocking platform of a precision scale. In parallel, 6% hydroxyethyl starch (Voluven^®^; Kabi-Frezenius) was infused through a 16-gauge peripheral catheter on the opposite arm, to a ratio of 1.15:1 to the donated blood.[7] The blood volume to be removed was calculated according to a standard formula to reach a hematocrit of 28%.[8] The whole-blood/colloid exchange procedure lasted 20 min (range 15 to 25 min), and it could be interrupted if there were signs of myocardial ischemia and/or unresponsive hypotension. The autologous blood was labeled and reinfused intraoperatively when the transfusion criteria were met.

Global hemodynamic data (mean arterial pressure, pulmonary capillary wedge pressure, central venous pressure, cardiac index, stroke volume index, and systemic vascular resistance index) were registered before the start of ANH and 5 min after ANH. All data were collected by trained observers who did not participate in patient care.

Statistical analysis was performed using the SigmaStat 2.03 software package (SPSS, Leuven, Belgium). Patient characteristics and hemodynamic parameters were compared between groups using unpaired Student's *t* test. Hemodynamic parameters were compared versus baseline using paired *t* test. Values are expressed as mean ± SD unless stated otherwise. Statistical significance was accepted at *P* < 0.05. All *P* values were two tailed.

## Results

Preoperative patient characteristics are summarized in Table 1. There was no significant difference between the two groups in any of the variables. Following blood withdrawal and isovolemic compensation with colloids, hemoglobin decreased from 14.5 ± 0.9 to 9.1 ± 1.0 (*P* < 0.001) in group A, and from 14.6 ± 1.1 to 8.9 ± 1.0 (*P* < 0.001) in group B. It was compensated by a slight increase in heart rate. However, central venous pressure and mean arterial pressure were within baseline values [Table 2]. No patients exhibited signs of myocardial ischemia as judged by the analysis of automated ST-segment and left ventricular wall motion monitoring.

**Table 1:** Preoperative Characteristics

Characteristics	Group A (n = 20)	Group B (n = 20)	*P* Value
Age, yr	57 (53-60)	56 (52-59)	0.8
BSA, m^2^	1.8 ± 0.2	1.9 ± 0.2	0.7
Sex (M/F)	16/4	15/5	0.8
Medical history		
Diabetes mellitus	5 (25)	5 (25)	1.0
Hypercholesterolemia	17 (85)	16 (80)	0.8
Hypertension	16 (80)	15 (75)	0.8
Medications			
ß-Blockers	17 (85)	18 (90)	0.8
Nitrates	9 (45)	8 (40)	0.8
ACE inhibitors	8 (40)	9 (45)	0.8
Calcium blockers	4 (20)	5 (25)	0.7
Diuretics	3 (15)	4 (20)	0.8
LVEF, %	56 (50-61)	54 (52-60)	0.8
left main stenosis ≥ 50%	4 (20)	5 (25)	0.7
Hb (g/dL)	14.5 ± 0.9	14.6 ± 1.1	
No. of bypasses			
(median [range])	4 (2-7)	4 (2-6)	

BSA = body surface area; LVEF = left ventricular ejection fraction; ACE = angiotensin-converting enzyme. Data are presented as mean (95% confidence interval) or No. (%) of patients unless otherwise indicated.

**Table 2:** Hemodynamic variables before and after acute normovolemic hemodilution

	Before ANH	After ANH
Hb (g/dL)		
Group A	14.5 ± 0.9	9.1 ± 1.0^†^
Group B	14.6 ± 1.1	8.9 ± 1.0^†^
Heart rate (beats/min)		
Group A	96 ± 5	99 ± 8
Group B	95 ± 6	100 ± 10
MAP (mmHg)		
Group A	80 ± 10	78 ± 8
Group B	81 ± 10	78 ± 11
CVP (mmHg)		
Group A	11.4 ± 1.9	11.6 ± 2.1
Group B	11.0 ± 2.0	11.2 ± 2.4
Cardiac output (L/min)		
Group A	5.07±1.17	5.02±1.28
Group B	4.84±1.21	6.02±1.28^†^
SVR (dyne*sec)/cm5		
Group A	1,089±187	1,042±198
Group B	1,162±144	884±137^†^

ANH = acute normovolemic hemodilution; CVP = central venous pressure; Hb = hemoglobin; MAP = mean arterial pressure; SVR = systemic vascular resistance. Data are presented as mean ± SD. ^†^*P* < 0.05 compared with before ANH, ^‡^*P* < 0.05 between groups

After ANH, systemic vascular resistance was slightly reduced from 1,089±187 to 1,042±198 mmHg/L/min in group A while there was a significant decrease from 1,162±144 to 884±137 mmHg/L/min in group B. In addition, the change in cardiac output was different between both groups. In group A, cardiac output was slightly decreased from 5.07±1.17 l/min to 5.02±1.28 l/min after ANH, whereas in group B, cardiac output was significantly increased from 4.84±1.21 l/min to 6.02±1.28 l/min after ANH.

## Discussion

The results of the present study demonstrated that in coronary surgery patients, with baseline heart rate faster than 90 bpm, anesthesia with sevoflurane during ANH was associated with an improvement in myocardial function after ANH, which was not present in patients anesthetized with midazolam. Acute normovolemic hemodilution does not per se reflect ischemic status of the myocardium. Several factors are known to determine occurrence of myocardial and other organ damage and outcome after coronary surgery. Among these, patient characteristics and surgery-related events are the common reasons for possible complications. The degree of stenosis in coronary arteries and myocardial area are also important. In addition, ANH in severe left main stem coronary artery disease has also greater significance compared to triple or double vessel disease. However, all patient characteristics, anesthetic depth, surgical and cardioprotective strategies were similar in both groups. This implies that the only difference between the groups was the choice of associated anesthetic drug: midazolam or sevoflurane.

The effects of acute normovolemic hemodilution (ANH) on myocardial function in patients with coronary artery disease are still not fully elucidated. Experimental and clinical evidence has indicated that volatile anesthetics have cardioprotective effects that are related to a preconditioning and a post-conditioning effect.[10–18] The use of a volatile anesthetic regimen might also have beneficial effects on cardioprotection during acute normovolemic hemodilution. The safety of the hemodilution procedure was ascertained by maintaining circulatory normovolemia and by close monitoring of cardiovascular parameters with ECG and echocardiography. Presumably, general anesthesia and chronic ß-blockade decreased the metabolic needs (approximately 20 to 30%) and prevented the sympathetic-mediated inotropic and chronotropic response.[19] Occasional reports of myocardial ischemia have been attributed to extremely low hemoglobin levels, concomitant hypovolemia, reflex tachycardia in awake volunteers, and/or increased postoperative metabolic needs.[20–22]

Hibernating myocardium results in recovery of myocardial function dramatically and the increase in cardiac output can be attributed to that. This also puts the severity of disease into perspective, as the decrease in cardiac output in midazolam group was not statistically significant. However, a previous study[7] indicated coronary surgery patients anesthetized with midazolam, pacing at 90 bpm during ANH were associated with depression of myocardial function which was not present in patients paced at 70 bpm. Our hypothesis is that the cardioprotective properties of a volatile anesthetic regimen[8,9] might also protect against the ANH related myocardial functional impairment in patients with baseline heart rate faster than 90 bpm. In the present study, cardiac output was slightly decreased after ANH in patients anesthetized with midazolam, whereas cardiac output was significantly improved after ANH in patients anesthetized with sevoflurane. The results of this study indicated that beside the slow baseline heart rate which was associated with an improvement in myocardial function after ANH, the use of a volatile anesthetic regimen could preserve myocardial function in patients with faster baseline heart rate. The limitation in this study was whether the chosen parameters were the optimal parameters to describe the whole myocardial function. Further experimental and clinical study using other parameters such as ejection fraction using transesophageal echocardiography should be investigated. In addition, the present study was limited until ANH, however the process of CPB and after-effects should have been analyzed until completion of surgery to give insights. However, according to cardioprotective strategies of our institute, during and after CPB, all patients were anesthetized with sevoflurane. So, we could not assess cardiac output changes during and after CPB.

In conclusion, in coronary surgery patients with baseline heart rate faster than 90 bpm, anesthesia with sevoflurane during ANH was associated with an improvement in myocardial function after ANH, which was not present in patients anesthetized with midazolam.
